# A Dietary Intervention in Adults with Overweight or Obesity Leads to Weight Loss Irrespective of Macronutrient Composition

**DOI:** 10.3390/nu16172842

**Published:** 2024-08-25

**Authors:** Maria Kafyra, Ioanna Panagiota Kalafati, Garyfallia Stefanou, Georgia Kourlaba, Panagiotis Moulos, Iraklis Varlamis, Andriana C. Kaliora, George V. Dedoussis

**Affiliations:** 1 Department of Nutrition and Dietetics, School of Health Science and Education, Harokopio University of Athens, 17671 Athens, Greece; nkalafati@gmail.com (I.P.K.); akaliora@hua.gr (A.C.K.); 2 Department of Nutrition and Dietetics, School of Physical Education, Sport Science and Dietetics, University of Thessaly, 42132 Trikala, Greece; 3 Biostatistics and Programming, ECONCARE, 11854 Athens, Greece; g_stefanou@hotmail.com; 4 Department of Nursing, University of Peloponnese, 22100 Tripoli, Greece; g.kourlaba@uop.gr; 5 Institute for Fundamental Biomedical Research, Biomedical Sciences Research Center ‘Alexander Fleming’, 16672 Vari, Greece; moulos@fleming.gr; 6 Department of Informatics and Telematics, School of Digital Technology, Harokopio University of Athens, 17778 Athens, Greece; varlamis@hua.gr

**Keywords:** obesity, weight loss, dietary intervention, macronutrient content, body weight management, nutrigenetics, lifestyle

## Abstract

Obesity is a critical public health issue, necessitating effective weight loss interventions. While various dietary regimens have been explored, individual responses to interventions often vary. This study involved a 3-month dietary intervention aiming at assessing the role of macronutrient composition and the potential role of genetic predisposition in weight loss among Greek adults. This randomized clinical trial followed the CONSORT principles, recruiting 202 participants overall; 94 received a hypocaloric, high-protein diet and 108 received a high-carbohydrate, hypocaloric diet. Genetic predispositions were assessed through 10 target variants known for their BMI associations. Participants’ weight and BMI values were recorded at baseline and post-intervention (*n =* 202 at baseline, *n* = 84 post-intervention) and an imputation method was applied to account for the observed missing values. Participants experienced a statistically significant weight loss across all dietary regimens (*p* < 0.001). Genetic analyses did not display statistically significant effects on weight loss. No significant differences in weight loss were observed between macronutrient groups, aligning with the POUNDS Lost and DIETFITS studies. This study underscores the importance of dietary interventions for weight loss and the potential contributions of genetic makeup. These findings contribute to obesity management within the Greek population and support the need for further research in personalized interventions.

## 1. Introduction

Obesity is a global public health challenge, with its prevalence rising at an alarming rate across diverse populations. The World Health Organization (WHO) states that 2.5 billion adults presented overweightness in 2022, with approximately 10% of those further presenting obesity [1]. According to the World Obesity Federation for the Greek area in 2019, approximately 50% and 17% of men presented overweightness and obesity, respectively, while the respective trends for women were 16.6% and 32.6% [2]. Obesity as a condition is a major risk factor for a range of chronic diseases, including type 2 diabetes, cardiovascular diseases, and certain cancers [3,4]. Addressing obesity effectively requires multifaceted strategies, among which dietary interventions play a critical role. Various dietary regimens, from low-carbohydrate to low-fat and balanced macronutrient compositions, have been proposed and studied extensively [5]. Interventions for weight management in Greece align with the ones proposed in the wider European area, where practices mainly focus on weight loss through hypocaloric or restrictive dietary regimens, exercise recommendations, anti-obesity medications, and several digital health initiatives [6,7]. However, the efficacy of these interventions often proves inadequate with success rates for weight loss and weight loss long-term maintenance varying widely among individuals [7] due to the complex interplay of genetic, environmental, and behavioral factors [8].

Recent advancements in nutrigenetics suggest that genetic predisposition can significantly impact individual responses to dietary interventions. Single nucleotide polymorphisms (SNPs) and gene–diet interactions have emerged as key areas of interest, offering potential pathways to personalized nutrition [9,10]. Despite these advancements, there remains a gap in understanding how these genetic factors interact with dietary interventions in different populations [8,10].

The assessment of gene–diet interactions in weight management is a necessity to deepen the understanding of the effects of genetic makeup on obesity onset. Current literature shows that modern practices incorporating long-distance tools for the delivery of dietary interventions can yield significant benefits for cardiometabolic profiles in the long run [9]. Currently, and to the best of our knowledge, there is no dietary intervention in the Greek population aiming to assess gene–diet interactions in the weight management of populations with obesity. 

This paper aims to explore the role of macronutrient composition and potential gene–diet interactions in weight loss among Greek adults, a demographic that has not been extensively studied in this context. The present study proposed adherence to a hypocaloric dietary regime of either high protein or high carbohydrate content for a period of three months with the overall aim of achieving weight loss in adults with overweightness or obesity. 

By leveraging both traditional and innovative online assessment tools, this study seeks to provide comprehensive insights into the effectiveness of various dietary regimens. Furthermore, it assesses the results of a long-distance dietary intervention in the context of the challenges surrounding the COVID-19 pandemic, particularly in terms of participant recruitment and adherence to dietary protocols. Through this research, we aim to contribute to a broader understanding of personalized dietary interventions and their implications for obesity management.

## 2. Materials and Methods

### 2.1. The iMPROVE Study Design and Ethical Considerations

The iMPROVE study concerned a dietary intervention for weight loss in adults with overweightness or obesity. Details of the study have been previously described elsewhere [11]. The information on the PICO (i.e., patient, population, intervention, comparison, and outcome) format of the study are described below. The study referred to adults with a body mass index (BMI) of above 25 km/m^2^, aged 18–65 years old, without accompanying and unregulated health comorbidities (i.e., patients) from the Greek population living in the Attica region from 2020 to 2021 (population). The intervention refers to a two-arm, parallel (comparison), randomized clinical trial for weight loss proposing adherence to one out of two different hypocaloric dietary regimens: one high in carbohydrates (60% of energy intake from carbohydrates, 18% protein, and 22% fat), and one high in protein (40% of the total energy intake from carbohydrates, 30% protein, and 30% fat), for a total period of three months (intervention and outcome). 

The primary expected outcome of the study was the beneficial effect of the intervention on weight loss and improvements in anthropometric indices, while also investigating the combined effect of genetic makeup. Secondary expected outcomes included the effect of the intervention on the enhancement of body composition, biochemical, and lifestyle parameters. The study was conducted according to the Declaration of Helsinki and was approved by the Ethical Review Board of Harokopio University of Athens, with a protocol number of 1800/13-06-2019. Healthcare professionals (i.e., dietitians and nutritionists) explained the study protocol to all participants, prior to the latter providing written consent before enrolling in the study. The study design has been registered to the ClinicalTrials.gov database of clinical studies (ClinicalTrials.gov Identifier: NCT04699448).

### 2.2. Study Participants and Intervention Characteristics

Inclusion criteria for the iMPROVE study participants referred to adults up to 65 years of age, with overweightness or obesity, and without accompanying and unregulated health comorbidities. Exclusion criteria included (i) pregnancy or lactation for women; (ii) diagnosed comorbidities affecting body weight and/or diet; (iii) intake of dietary supplements pertaining to weight loss; and (iv) simultaneous research participation for weight management and/or weight loss [12]. Overall, 202 volunteers were recruited in Harokopio University from 2020 to 2021. The total sample size was calculated based on the desired power for achieving statistically significance in the reduction of BMI across the two parallel arms [12]. 

Details of the intervention characteristics have been previously provided elsewhere [12]. Briefly, after inclusion in the study, participants were randomly allocated to either the high-carbohydrate or the high-protein hypocaloric diet through simple randomization. Daily dietary intake for each participant was reduced by 500 kcal, as the optimal target for weight loss aims at 0.5 to 1 kg per week, i.e., a 7500 kcal weekly caloric reduction, or a 500 kcal daily caloric reduction [11]. A hypocaloric diet with either high protein or high carbohydrate content was subsequently administered to each participant via an online assessment tool developed by the team in Harokopio University. 

During the baseline session, the nutrition expert provided each volunteer with a virtual tour of the online assessment tool, as well as unique usernames and passwords to access it. Then, participants were required to complete a series of questionnaires to provide information on their medical history; demographic details; satiety levels; adherence to the Mediterranean dietary pattern; depression symptoms; quality of life and health status; sleep quality; as well as dietary and physical activity habits [12]. Participants would find and download the proposed diets and self-report anthropometric measurements and lifestyle characteristics through the completion of the online questionnaires at the end of each intervention month. Thus, the participant would receive the proposed diet through access to the platform, which was updated monthly after the nutrition expert reviewed the questionnaires from the previous month [12]. Healthcare professionals (dietitians and nutritionists) regularly monitored participants’ progress [11] and renewed the hypocaloric diet at the end of each month, according to each participant’s progress (i.e., re-adjusting caloric intake according to the kg lost per month). The present article describes the results of the intervention at 3 months after baseline on anthropometric and body composition indices.

### 2.3. Anthropometric Measurements and Body Composition Analysis

Collection of the anthropometric data during the in-person meetings at baseline and the end of month 3 included height (measured to the nearest 0.1 cm), weight (measured to the nearest 0.1 kg), and waist and hip circumferences. Height was measured with the participants barefoot and using a portable stadiometer. Weight was measured with participants wearing light clothing, using the Tanita BC-418 Segmental Body Composition Analyzer, Tanita, Tanita Europe (Amsterdam, The Netherlands), while waist and hip circumference (WC and HC) measurements took place between the twelfth rib and the iliac crest and at the widest point of the hips, respectively, using a non-extensible soft tape, and the waist-to-hip ratio (WHR) was calculated by dividing the WC by the HC. Body composition analysis took place via a bioelectrical impendence analysis machine (Tanita Body Composition Analyzer BC-418), where the participants wore light clothing and did not carry any metal objects. Participants were informed not to consume any food or drink and not undergo mediocre or intense physical activity/exercise for at least 2 h prior to the analysis. Body composition analysis provided data on the individual’s ΒΜΙ, total and departmental body fat, as well as muscle and water percentages.

### 2.4. Laboratory Analyses

Blood samples (23 mL) were collected from each participant in all in-person meetings, following a 12-hour overnight fast. Blood samples were collected from each participant during daytime until 10.30 am, two of them in EDTA blood collection tubes and one in a non-EDTA-containing Vacutainer. The buffy coat samples collected after centrifuging of the blood samples were used for DNA extraction. Part of the samples were extracted using the Invitrogen iPrep Purification Instrument, Thermo Fischer Scientific SA (Waltham, MA, USA) and the Invitrogen iPrep PureLink gDNA Blood Kit, Thermo Fischer Scientific SA [12], while DNA extraction also took place manually for a small part of the samples using PureLink^®^ Genomic DNA, Thermo Fischer Scientific SA kits. Quantification of the extracted DNA took place via use of a spectrophotometer. All DNA samples were then stored at −20 °C for a maximum duration of 2 months up to being sent for further analyses and genotyping. If storage was needed for more than 2 months, samples were stored at −80 °C. Genotyping of at least 50 ng/μL of the extracted DNA samples took place via (i) use of the Axiom Precision Medicine Diversity Research Array (PMD Research Array), containing more than 800,000 SNPs, deletions, and copy number variations from the 1000Genomes Project Phase III [13], for a part of the extracted samples [14]; (ii) use of the Axiom Precision Medicine Diversity Research Array (PMRA), containing more than 800,000 markers for different groups of extracted samples [15]; and (iii) the Illumina Global Screening Array [16]. Following completion of the genotyping, imputation analyses took place using the 1000 genomes Phase 3 panel and IMPUTE version 2 software [17]. 

### 2.5. Effect of Candidate Variants on Post-Intervention Changes

To investigate the impact of genetic predisposition on the indices examined at baseline and post-intervention, we approached the assessment of the effect of candidate genetic variants by analyzing 10 specific variants known for their associations with BMI, based on current literature and the availability of study data at the time of analyses [18] (Table 1). Multiple FTO variants were examined due to the existing literature behind the relation of multiple FTO SNPs and weight management. We used a threshold of 0.8 for the imputation INFO score for all SNPs included in the analyses. Quality control for sample and SNP exclusion criteria consisted of (i) a sample call rate of 95%; (ii) a genotyping call rate of 98%; (iii) a Hardy–Weinberg Equilibrium (HWE) exact test at *p* < 0.0001; and (iv) a minor allele frequency at 1%.

### 2.6. Statistical Analyses

For the present analyses and regarding the baseline measurements, we employed the data collected during the in-person meeting, whereas data provided by the participants at the end of each month up to 3 months were used to assess changes from baseline, using the collected data. More specifically, for the end of month 3, we used the in-person measurements for the participants who attended the in-person meeting and the online, self-reported measurements for the participants who did not attend the in-person session. 

Data analyses were conducted using the Statistical Package for Social Sciences (SPSS), version 23 [19], the R statistical version 4.2.0. [20], and STATA statistical software version 17 [21]. The Shapiro–Wilk and Kolmogorov–Smirnov tests and Q-Q plots were used to evaluate the distributions of the variables. For variables following a normal distribution, mean values and standard deviation are presented, while the medians and interquartile ranges are presented for variables that are not normally distributed. The non-parametric Mann–Whitney test was used to assess differences between the two sexes and diet groups and the non-parametric Wilcoxon signed-rank test to assess differences pre- and post-intervention, as well as differences between baseline and the end of each examined month. Multivariate linear regressions were employed to test for potential associations between the various phenotypic variables and are presented as beta coefficients (β) and standard error (SE). Variables not following the normal distribution were log transformed. 

### 2.7. Imputation of Missing Values

Due to the high number of missing values in the anthropometric measurements, an imputation methodology was used to fill in the missing weights observed at the end of three months. This imputation was performed on the logarithm of weight at the end of the first, second, and third months using a multivariate normal regression model.

During the imputation process, the effects of various variables on the primary outcomes were examined, including age, sex, physical activity, baseline anthropometric measurements (body fat percentage, waist circumference, and visceral fat), baseline biochemical indices (total cholesterol, glucose, triglycerides, HDL-C), and socio-economic factors (family status and education level). Family status data were analyzed to determine each participant’s living situation (living alone if single, separated, divorced, or widowed; not living alone if married or in a steady relationship). Education level was categorized as no education, completion of 1st, 2nd, or 3rd grade, or other reported characteristics. Correlation analyses assessed the effect of numerical variables (body fat percentage, waist circumference, visceral fat, glucose, triglycerides, HDL-C) on baseline weight and weight change at 3 months.

To address post-intervention missing values, STATA software was used for imputation via the multiple imputation method, with three steps: (i) generating imputed datasets; (ii) performing the desired analysis separately on each dataset; and (iii) combining the results into a single model. The missing data were assumed to be missing at random (MAR), as follow-up loss did not differ between diet groups and no other variables correlated with follow-up loss. Sensitivity analyses verified the stability of the inferences. Correlations between baseline weight and weight change at 3 months with categorical variables were assessed using Student’s *t*-test, the Mann–Whitney test, and the Kruskal–Wallis test, while correlations with numerical variables were assessed using Spearman’s rho.

The imputation step covered body weight at the 1st, 2nd, and 3rd months using a multivariate normal regression model (multiple variables with missing values were all + imputed together). Due to the non-normal distribution of weight, the logarithm of weight was imputed for different scenarios, testing for key anthropometric and lifestyle variables: (i) Weight Baseline, Sex, Age, Diet Group; (ii) Weight Baseline, Sex, Age, Diet Group, Live Alone; (iii) Weight Baseline, Sex, Age, Diet Group, Live Alone, Education Years; and (iv) Base Case: Weight Baseline, Sex, Age, Diet Group, Live Alone, Fat (%) Baseline. For each scenario, 20, 50, and 100 imputations were performed, with robust results from the latter. The imputation used a multivariate normal regression model with an iterative Markov Chain Monte Carlo (MCMC) procedure. A burn-in period of 10,000 iterations, 1000 iterations between 100 imputations, and a non-informative prior distribution (Jeffreys) were assumed. Model convergence was checked using trace and autocorrelation plots.

For each of the 100 datasets, the logarithm of weight was converted back to its normal scale for each month. The weight change was computed as the difference between the weight at the 3rd month and the baseline weight. Convergence was verified with trace and autocorrelation plots. In the pooling step, a null linear regression model estimated the mean (95% CI) change in body weight at 3 months for the total sample. An additional linear regression model compared the mean weight change at 3 months between the two diet groups, with results presented as beta coefficients (95% CIs).

## 3. Results

### 3.1. Study Enrolment 

**Figure 1:**
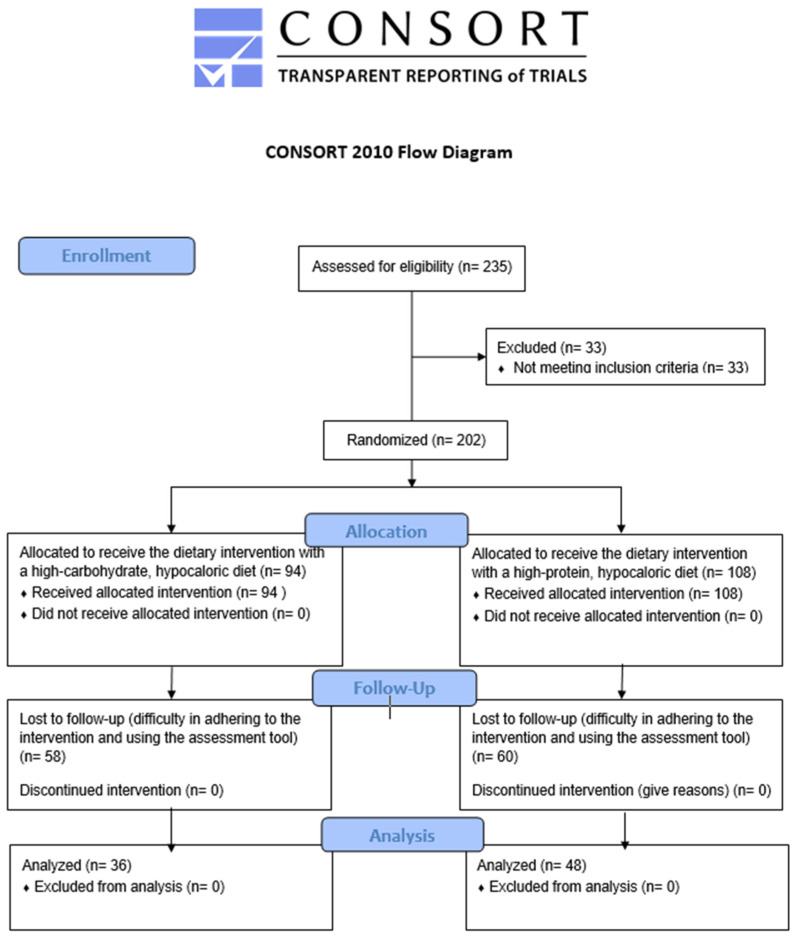
CONSORT 2010 flow diagram for the iMPROVE study.

Overall, 235 volunteers were screened for eligibility to enter the study in a continuous way (i.e., first subject in—first subject out), 33 of whom were excluded due to not meeting the inclusion criteria. Ultimately, 202 participants were allocated to one of the two hypocaloric regimen groups; 94 participants to the high-carbohydrate group and 108 participants to the high-protein group. The safety of the participants was continuously ensured via the dynamic monitoring and the re-adjustment of the administered diets based on the participants’ evolving needs. By the 3-month follow-up, 58 participants from the former and 60 participants from the latter group had dropped out (61.7% and 55.55%, respectively) due to difficulty adhering to the hypocaloric diet, and the percentage of lost-to-follow-up did not significantly differ between the two dietary groups. As a result, 84 participants (36 from the high-carbohydrate group and 48 from the high-protein group) attended the end-of-month-3 in-person meetings, providing data to be used for the subsequent analyses. Figure 1 depicts the CONSORT 2010 diagram [22] for the iMPROVE study recruitment process.

### 3.2. Changes Observed in Collected Data for Weight and BMI in the Overall Sample

We first used the collected data (overall sample of the 84 participants attending the end-of-month-3 in-person meetings) to assess changes from baseline for weight and BMI. In the entirety of the sample, both indices noted a statistically significant decrease at the end of month 3 compared to baseline (83 kg vs. 87 kg, *p* < 0.001 and 29.32 kg/m^2^ vs. 31.35 kg/m^2^, *p* < 0.001, respectively), as shown in Table 2, showing a statistically significant effect size of the intervention on the primary outcomes, i.e., weight loss and subsequent BMI change. Both men and women showed significantly lower levels of BMI and weight in month 3 (*p* < 0.001 for all), with men noting a median decrease of 5 kg and women showing a decrease of 6 kg from baseline. Table 2 further summarizes the changes observed in weight and BMI per month of the intervention, where a statistically significant decrease in weight and BMI from baseline up to the end of month 1 was observed (87 kg vs. 84 kg, *p* < 0.001 and 31.35 kg/m^2^ vs. 30.14 kg/m^2^, *p* < 0.001, respectively). Similarly, the indices were statistically significantly lower from the end of month 1 up to the end of month 2 (84 kg vs. 82 kg, *p* < 0.001 and 30.14 kg/m^2^ vs. 29.71 kg/m^2^, *p* < 0.001, respectively). However, the changes observed from the end of month 2 up to the end of month 3 did not show statistical importance. Similarly to above, the changes observed were not differentiated across the two sexes, with both men and women showing statistically significant decreases from baseline to the end of month 1 and from the end of month 1 to the end of month 2, but not from the end of month 2 to the end of month 3.

### 3.3. Changes Observed in Collected Data for Weight and BMI across the Two Diet Groups

As far as the changes observed within the two different diet groups are concerned, BMI and weight changes were not statistically significantly differentiated between the two groups (Table 3), as participants in both groups showed statistically significant reductions in BMI and weight (*p* < 0.001 for all). 

Regarding the within-diet-group changes per month of the intervention, weight was statistically significantly reduced from baseline to month 1 and month 1 to month 2 for participants in both groups (*p* < 0.05 for all) (Table 3). Additionally, BMI levels decreased for both groups from baseline to the end of month 1 (*p* < 0.001 for both), whereas participants in the high-protein groups also displayed a statistically significant reduction from the end of month 1 up to the end of month 2 (31.18 kg/m^2^ vs. 29.71 kg/m^2^, *p* = 0.006). 

### 3.4. Results from Imputation of Missing Values

Following the data analyses using the collected data, we proceeded to applying the imputation methodology to account for the missing weight values of the participants at the end of month 3, selecting key anthropometric and lifestyle variables, as described in the Methodology section. The weight change observed among the participants in the two categories of living status showed statistically significant differentiations, where individuals who were reported to be living alone noted lower change compared to the ones living with someone else (change = −1 kg vs. change = −3 kg, *p* = 0.003). Table 4 illustrates the comparisons between each categorical value group examined and the observed change in weight at month 3. Among the numerical variables, body fat percentage (*p* = 0.007), WC, visceral fat, glucose, TG, and HDL-C levels were correlated to baseline weight (*p* < 0.001), but only visceral fat was associated with the change in weight at the end of the three months (Table 5).

In order to finalize the variables to be included in the imputation process, four different scenarios were examined including (i) scenario 1, consisting of weight at baseline, sex, age, and diet group; (ii) scenario 2, consisting of weight at baseline, sex, age, diet group, and living status; (iii) scenario three consisting of weight at baseline, sex, age, diet group, living status, and education years; and (iv) the “base case” scenario consisting of weight at baseline, sex, age, diet group, living status, and body fat percentage at baseline (Table 6). 

Taking all participants into account, all four of the examined scenarios displayed a statistically significant reduction in weight, with the base case scenario presenting a mean −2.68 kg (*p* < 0.0001) reduction (Table 6). Furthermore, a linear regression model was fitted to assess potential differences in the 3-month body weight change between the two diet groups, which did not display any statistically significant differentiations (0.05 kg and 0.03 for the high-carbohydrate and protein groups, respectively). Although statistically insignificant, the mean difference between the two diet groups was −0.02 kg in favor of the high-protein group (*p* = 0.481) (Table 7). 

### 3.5. Effect of Candidate Variants on Weight Loss

**Figure 2:**
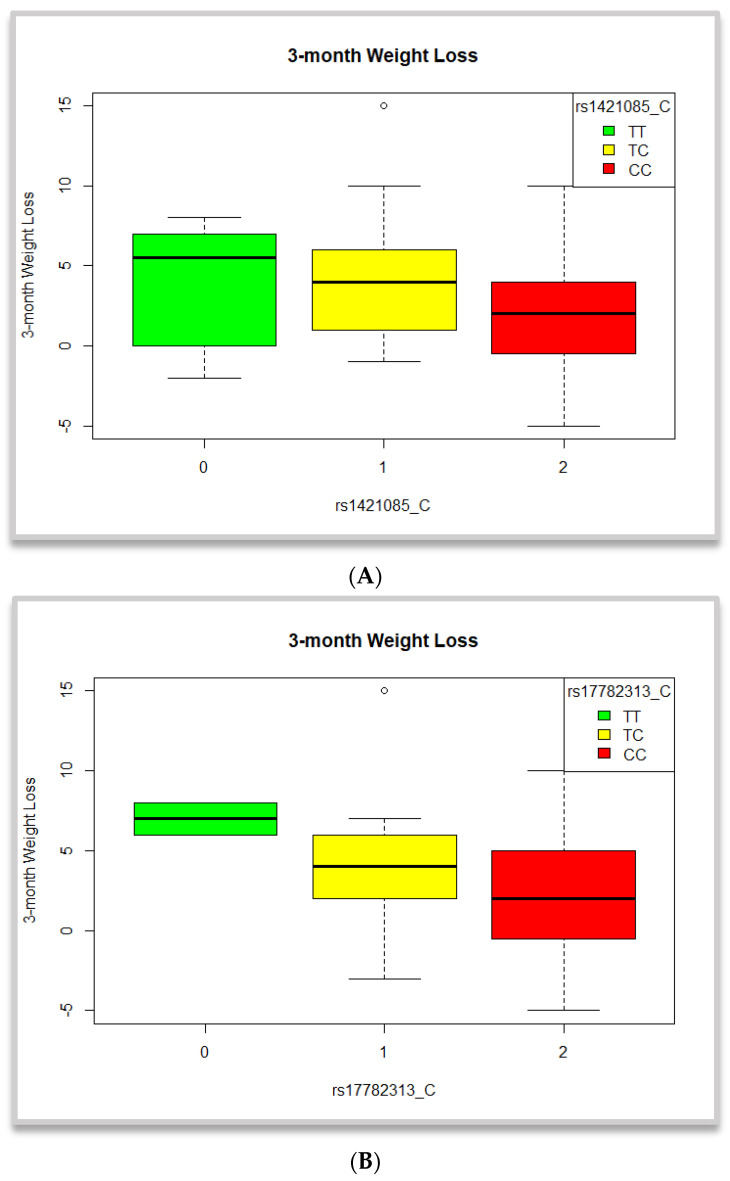
Clustered boxplots depicting 3-month weight loss (**A**) per genotype groups of the rs1421085 SNP and (**B**) per genotype groups of the rs17782313 SNP. Results are not significant.

Using the aforementioned, 10 BMI-related SNPs, Kruskal–Wallis tests revealed statistically significant differences in the observed weight change across the groups of the rs1421085 and the rs17782313 variants (Table 8). Carriers and mostly homozygotes for the BMI-raising C allele of the former displayed significantly lower change in weight post-intervention (*p* < 0.036, Figure 2). Similarly, carriers of the BMI-positively associated C allele of the rs17782313 SNP also showed significantly lower weight changes after the 3-month period (*p* = 0.043, Figure 2). 

We further used the previously described 10 BMI-related SNPs to examine potential associations with post-intervention weight change, using the imputed weight data. The SNPs did not display statistically significant associations for the observed weight change post-intervention. Within-diet group analyses also showed no statistically significant relations for weight loss in participants following either the high-carbohydrate or the high-protein diet (see Supplementary Table S1).

## 4. Discussion

The iMPROVE study concerned a dietary intervention that investigated the effects of two hypocaloric diets with different macronutrient content on anthropometric, biochemical, and lifestyle changes in adult participants with overweightness or obesity. Overall, 83 out of the 202 recruited participants completed the intervention at the end of month 3. Participants did not present uncontrolled accompanying disorders which could disrupt the effect of the proposed intervention on the expected outcomes. An imputation analysis for missing values took place to better assess the effects of the intervention at the end of the 3-month period. Statistically significant decreases in weight were observed (mean reduction of 2.68 kg, *p* < 0.0001 for all participants), without differentiations across the two diet groups. The reasons for this outcome lie (i) in the provision of the hypocaloric diet, which can directly result in weight loss; (ii) in sufficient adherence to the intervention by the participants due to the continuous monitoring and adapting based on the provided feedback; and (iii) with a sufficient time period for the intervention delivery that allowed for differences to be observed. Furthermore, this finding serves as the foundation to subsequently explore the potential implications on the amelioration of body composition and cardiometabolic profile indices, which have been previously described as secondary expected outcomes.

Additionally, in an effort to assess the potential role of genetic makeup, the effects of 10 candidate variants previously associated with BMI were investigated, based on genetic data availability at the time of analyses. Although within genotype groups the tests revealed differentiations between carriers of the risk alleles for FTO rs1421085 SNP and the MC4R 17782313 SNP when using the collected data for the 83 participants, linear regressions using the imputed data for the 202 participants did not show any statistically significant effects. 

Previous research on the impact of different macronutrient compositions for weight loss has not shown significant differences between diets with higher carbohydrate versus higher protein content. Consistent with our results, the POUNDS Lost trial reported similar outcomes for the 345 participants with both pre- and post-intervention data [23]. The trial had a comparable drop-out rate to that of the iMPROVE participants, with 42.55% of participants completing the POUNDS Lost trial (811 individuals with baseline data vs. 345 individuals with data at both baseline and the end of the 6-month period) and 41.58% completing the iMPROVE trial (202 individuals with baseline data vs. 84 individuals with data at both baseline and the end of the 3-month period). Compared to iMPROVE, POUNDS Lost noted nearly double the weight loss over double the intervention period, with a mean weight loss of 6 kg at the end of 6 months. Similar to the iMPROVE trial, no differences in weight loss were observed among the four dietary groups [23,24]. Additionally, another similar initiative in the context of the DIETFITS trial also showed no statistically significant differences in weight loss between the healthy low-fat and healthy low-carbohydrate groups at the end of the 12-month intervention period [25].

Parr et al. also investigated the effect of adhering to a 4-month hypocaloric diet, showing similar results to those of the present study [26]. More specifically, the study examined three groups with different macronutrient content [a high protein, moderate carbohydrate content (30% fat, 30% protein and 40% carbohydrate); a high protein, high carbohydrate content (15% fat, 30% protein, 55% carbohydrate), or a control regimen (30% fat, 15% protein and 55% carbohydrate], noting a weight loss of 7.7 kg in 89 adults with overweightness or obesity after the 4-month intervention period, without any differences observed in the changes within the examined diet groups [26]. Additionally, the NUGENOB project also investigated the effect of a 10-week hypocaloric diet of either low fat content (i.e., 20–25% fat, 15% protein, ~60 to 65% carbohydrate) or high fat composition (i.e., ~40 to 45% fat, 15% protein, ~40–45% carbohydrates) in the observed weight loss of 771 adults with obesity. Similarly to the iMPROVE study, the NUGENOB project results also showed no statistically significant interactions between the suggested diet groups and the weight loss outcome [27].

In the same trial, Handjieva-Darlenska et al. aimed to further assess the rate of weight loss throughout the intervention period [28]. They discovered that participants who lost less than 4 kg by the midpoint of the 10-week trial were likely to achieve a lower overall weight loss by the end of the study [28]. In our current study, we observed that changes in primary outcomes (i.e., weight and BMI) remained statistically significant up to the end of the second month (*p* < 0.05). This suggests that dietary interventions might be most effective when implemented over shorter periods when participants’ motivation is at its highest. Supporting this, a 2020 systematic review by Ge et al. found that both low-carbohydrate and low-fat diets produced similar weight loss results over a standard 6-month period, but the effect diminished by 12 months, regardless of the diet followed [29].

We further used the observed data to investigate potential differentiations in weight change across genotype groups. A statistically significant difference was shown between the three groups of genotypes for the FTO rs1421085 and the MC4R rs17782313 variants and change in body weight (*p* = 0.036 and *p* = 0.043, respectively), but was not maintained when using the imputed sample size. This could be potentially attributed to the increase in sample size and, thus, the increased heterogeneity and variance, as well as other unknown biases. However, this suggested effect of the two SNPs on increased weight has been previously mentioned in the literature, prompting us to explore their potential influence on weight loss. Franzago et al. studied the impact of five specific SNPs, including the FTO rs9939609 SNP and the MC4R rs17782313 variant, on weight loss in obese patients undergoing a nutritional intervention [30]. Their findings indicated that individuals with the rs9939069-A allele experienced a smaller reduction in BMI from baseline to the end of the 12-month intervention [30]. Although the present study showed no associations for the former SNP, said investigations might be affected by the restricted sample size examined.

Thus far, other dietary interventions aimed at weight loss have produced results similar to those of the iMPROVE trial, with most studies examining and reporting the impact of candidate polymorphisms or genetic risk scores of numerous selected variants on outcomes of interest. For instance, the DIETFITS trial, which included 609 overweight adults, found no statistically significant associations between gene–diet interactions and weight loss after the 12-month intervention period [26]. Similarly, the previously mentioned NUGENOB project, which investigated the potential effect of 42 candidate SNPs in 648 participants, reported only nominally significant associations for lower weight loss in individuals with the effect alleles who followed the low-fat diet [27]. 

### Strengths and Limitations

Overall, the present study has several significant advantages, being the first dietary intervention to examine the role of macronutrient composition and genetic background on weight loss in Greek adults. This study is the first of its kind in a Greek adult population, providing valuable insights into the associations between genetic predisposition and anthropometric and lifestyle factors. An additional innovation of the study referred to the use of an online assessment tool to facilitate long-distance communication and monitoring during the COVID-19 pandemic. This provides the first evidence for self-monitoring during a dietary intervention in Greek adults, allowing for the development of further research using these data to ameliorate such long-distance practices. In addition, the heterogeneity of the results should be seen as a strength, as it sets the stage for future research, aligning with the methods and findings of larger initiatives like the POUNDS Lost and the DIETFITS trials.

However, significant limitations were also noted, namely (i) the limited sample size due to the impact of the pandemic on volunteer recruitment, influenced by social distancing protocols and reduced interest in participation; (ii) the challenges surrounding the requirement for long-term adherence to a long-distance dietary intervention with extended periods between in-person follow-up meetings; (iii) the difficulty of older adults in the use of the online assessment tool due to limited access and knowledge of modern technological devices and online tools; and (iv) the use of limited candidate variants to assess genetic information. 

## 5. Conclusions

In summary, this is the first study investigating a hypocaloric intervention with different macronutrient contents using a long-distance online platform in Greek adults. The study showed significant weight loss by the end of the intervention period but without differences across the two dietary groups, underscoring the complexity and variability of dietary interventions for weight loss. With regards to the effect of genetic predisposition, available genetic data were used to highlight the nuanced interplay between diet and genetic predisposition, where carriers of the FTO rs1421085-A and the MC4R rs17782313-C alleles showed increased tendencies for weight loss but without large effect sizes in the examined population. 

While the present findings align with larger studies such as POUNDS Lost and DIETFITS, they also emphasize the unique context of the Greek population, contributing valuable insights to the global discourse on obesity management. Moving forward, future research should continue to investigate the multifaceted influences on weight loss, incorporating larger and more diverse cohorts and assessing genetic makeup via use of polygenic risk scores to refine our understanding of effective dietary interventions. By embracing a holistic perspective that considers genetic, environmental, and behavioral factors, we can enhance the efficacy of weight loss programs and ultimately improve public health outcomes.

## Figures and Tables

**Table 1:** List of the BMI-related SNPs (n = 10) investigated for associations in the iMPROVE cohort.

Consortial Summary Statistics								iMPROVE Cohort	
SNP	Gene	Chr	Position (bp)	Alleles	MAF	Effect Allele	Direction of Effect for BMI	MAF	Ref
rs6548238_T	LINC01875, TMEM18	2	2:634905	T/C/G	0.12 (T)	C	Positive	T: 0.095	GWAS Catalog
rs1801282_G	PPARG	3	3:12351626	C/G	0.07 (G)	G	Positive	G: 0.053	GWAS Catalog
rs2241766_G	APIPOQ	3	3:186853103	T/A/C/G	0.15 (G)	G	Positive	G: 0.45	GWAS Catalog
rs925946_T	BDNF	11	11:27645655	T/A/C/G	0.25 (T)	T	Positive	T: 0.103	GWAS Catalog
rs1421085_C	FTO	16	16:53767042	T/C	0.23 (C)	C	Positive	C: 0.26	GWAS Catalog
rs1121980_A	FTO	16	16:53775335	G/A/C	0.37 (A)	A	Positive	A: 0.28	GWAS Catalog
rs17817449_G	FTO	16	16:53779455	T/A/G	0.31 (G)	G	Positive	G: 0.28	GWAS Catalog
rs3751812_T	FTO	16	16:53784548	G/T	0.22 (T)	T	Positive	T: 0.25	GWAS Catalog
rs9939609_A	FTO	16	16:53786615	T/A	0.34 (A)	A	Positive	A: 0.26	GWAS Catalog
rs17782313_C	MC4R	18	18:60183864	T/A/C	0.24 (C)	C	Positive	C: 0.13	GWAS Catalog

BMI: Body Mass Index, SNP: Single nucleotide polymorphism, Chr: chromosome, bp: base pairs, MAF: minor allele frequency (as shown in GWAS Catalog), Ref: reference.

**Table 2:** Weight and BMI during the three months of the study.

Variable	Time	Total		Men		Women		
		*n*	Median (IQR)	*n*	Median (IQR)	*n*	Median (IQR)	*p* *
Weight	Baseline	202	87 (26)	59	103 (30)	143	83 (17)	<0.001
	Month 1	118	84 (25)	35	101 (21)	83	79 (15)	<0.001
	*p* **		<0.001 *		<0.001		<0.001	
	Month 2	89	82 (25)	27	100 (25)	62	76.50 (13)	<0.001
	*p* **		0.001 *		0.009		0.023	
	Month 3	84	83 (23)	25	98 (27.5)	59	77 (16)	<0.001
	*p* **		0.819 *		0.925		0.849	
	*p* ***		<0.001		<0.001		<0.001	
BMI	Baseline	202	31.35 (6.9)	25	29.63 (7.46)	58	28.93 (5.82)	0.920
	Month 1	118	30.14 (6.08)	35	31.13 (7.51)	83	29.73 (5.87)	0.528
	*p* **		<0.001		<0.001		<0.001	
	Month 2	89	29.71 (6.12)	27	31.01 (7.42)	62	29.33 (5.33)	0.215
	*p* **		0.001		0.010		0.044	
	Month 3	84	29.43 (6.50)	25	29.63 (7.46)	59	28.98 (5.79)	0.333
	*p* **		0.867		0.955		0.670	
	*p* ***		<0.001		<0.001		<0.001	

BMI: Body Mass Index, IQR: Interquartile Range. *: *p*-value showing differences within the two sexes, using the Mann–Whitney test. **: p-value showing overall change from the previous month using the Wilcoxon signed-rank test. ***: *p*-value showing overall change from baseline using the Wilcoxon signed-rank test.

**Table 3:** Changes in weight and BMI during the three months of the study, per diet group.

Variable	Time	*n1* (High Carb)	Median (IQR)	*n2* (High Prot)	Median (IQR)	*p* *
Weight	Baseline	94	83.50 (26)	108	88.50 (25)	0.014
	Month 1	56	81.50 (21)	62	86 (26)	0.173
	*p* **		<0.001		<0.001	
	Month 2	42	80 (20)	47	86 (25)	0.149
	*p* **		0.047		0.006	
	Month 3	36	79 (25)	48	84.5 (20.5)	0.178
	*p* **		0.478		0.843	
	*p* ***		<0.001		<0.001	
BMI	Baseline	94	30.5 (6.9)	108	32.3 (7.8)	0.920
	Month 1	56	29.58 (6.34)	62	31.18 (6.09)	0.249
	*p* **		<0.001		<0.001	
	Month 2	42	29.84 (5.43)	47	29.71 (7.02)	0.421
	*p* **		0.088		0.006	
	Month 3	36	29.21 (6.83)	48	29.47 (6.32)	0.333
	*p* **		0.458		0.610	
	*p* ***		<0.001		<0.001	

BMI: Body Mass Index, IQR: Interquartile Range. *: *p*-value showing differences within the two diet groups, using the Mann–Whitney test. **: *p*-value showing change from the previous month for each diet group, using the Wilcoxon signed-rank test. *** *p*-value showing overall change from baseline using the Wilcoxon signed-rank test.

**Table 4:** Comparison of baseline weight and change in weight at 3 months across the categories of possible categorical predictors.

	Baseline Weight, kg			Change in Weight at 3 Months, kg		
	*n*	Descriptives	*p*	*n*	Descriptives	*p*
Sex		Median (Q1, Q3)			Mean (SD)	
Male	59	103 (90, 120)	<0.001 *	25	−4.22 (3.96)	0.250 **
Female	143	83 (75, 92)		59	−2.47 (3.29)	
Physical activity		Median (Q1, Q3)			Median (Q1, Q3)	
Sedentary	64	88 (79, 105)		22	−1 (−4, 1)	
Mediocre	104	86 (77.5, 100)	0.369 ***	49	−3 (−6, 0)	0.197 ***
Active/intense	31	88 (81, 104)		12	−6.75 (−4.5, 0)	
Live alone		Median (Q1, Q3)			Mean (SD)	
Yes	59	86 (74, 104)	0.231 *	21	−1.05 (3.01)	0.003 **
No	142	88 (78, 104)		62	−3.70 (3.52)	
Education level		Median (Q1, Q3)			Median (Q1, Q3)	
No education	8	94.5 (84, 97.5)		6	−5.75 (−6, −3.5)	
1st grade	2	95 (86, 104)		1	3.5 (3.5, 3.5)	
2nd grade	54	87 (78, 110)	0.664 ***	22	−2.5 (−4.5, −1)	0.382 ***
3rd grade	125	86 (77, 103)		50	−3 (−6, 1)	
Other	12	86.5 (80, 90)		4	−3.5 (−6.5, 0)	

SD: Standard Deviation, Q1: Quartile 1, Q3: Quartile 3. *: *p*-value showing differences within the two groups, using the Mann–Whitney test. **: *p*-value showing differences within the two groups, using Kruskal–Wallis test. ***: *p*-value showing differences within the two groups, using Student’s *t*-test.

**Table 5:** Correlation of baseline weight and change at 3 months with possible numerical predictors.

	Correlation with Baseline Weight			Correlation with Change in Weight at 3 Months		
	N	Spearman’s Rho	*p*	N	Spearman’s Rho	*p*
Age	202	−0.04	0.563	84	−0.09	0.413
Body Fat %	202	0.19	0.007	84	0.16	0.149
WC	183	0.75	<0.001	77	−0.18	0.114
Visceral fat	202	0.79	<0.001	84	−0.22	0.049
Total cholesterol	193	−0.07	0.320	83	0.11	0.314
Glucose	193	0.25	<0.001	83	−0.10	0.388
TG	193	0.31	<0.001	83	−0.01	0.906
HDL-C	193	−0.37	<0.001	83	0.14	0.213

WC: Waist Circumference, TG: Triglycerides, HDL-C: High Density Cholesterol.

**Table 6:** Mean changes in weight for overall population and mean differences between diet groups at 3 months (in kg).

*n* = 202				Change in Weight at 3 Months (kg)		Crude Difference between Diet Groups *	
		M	*n*	Mean (95% CI)	*p*	b (95% CI)	*p*
CCS		0	84	−2.99 (−3.77, −2.22)	<0.0001	0.23 (−1.34, 1.80)	0.773
	**Covariates used for imputation**						
Sc.1	Weight Baseline, Sex, Age, Diet Group	100	202	−2.58 (−3.59, −1.57)	<0.0001	0.36 (−1.54, 2.26)	0.704
Sc.2	Weight Baseline, Sex, Age, Diet Group, Live alone	100	202	−2.64 (−3.56, −1.72)	<0.0001	0.36 (−1.39, 2.11)	0.682
Sc.3	Weight Baseline, Sex, Age, Diet Group, Live alone, Education years	100	185	−2.54 (−3.56, −1.53)	<0.0001	0.31 (−1.72, 2.34)	0.762
Base case	Weight Baseline, Sex, Age, Diet Group, Live alone, Fat (%) baseline	100	202	−2.68 (−3.55, −1.80)	<0.0001	0.39 (−1.29, 2.06)	0.647

b: beta, CCS: Complete case scenario; M: # of imputations * linear regression model of weight difference at 3 months. Coefficients (weight difference) presented for diet group (high protein vs. high carbohydrate).

**Table 7:** Mean changes in weight by diet group at 3 months (in kg).

*n* = 202	Change in Weight at 3 Months (kg)	High Protein			High Carbohydrate		
		*n*	Mean (95% CI)	*p*	*n*	b (95% CI)	*p*
CCS		48	−2.90 (−4.03, −1.76)	<0.0001	36	−3.13 (−4.18, −2.07)	<0.0001
	**Covariates used for imputation**						
Sc.1	Weight Baseline, Sex, Age, Diet Group	108	−2.41 (−3.81, −1.02)	0.001	94	−2.77 (−4.19, −1.36)	<0.0001
Sc.2	Weight Baseline, Sex, Age, Diet Group, Live alone	108	−2.47 (−3.75, −1.19)	<0.0001	94	−2.83 (−4.12, −1.55)	<0.0001
Sc.3	Weight Baseline, Sex, Age, Diet Group, Live alone, Education years	98	−2.40 (−3.82, −0.98)	<0.0001	87	−2.71 (−4.20, −1.22)	<0.0001
Base case	Weight Baseline, Sex, Age, Diet Group, Live alone, Fat (%) baseline	108	−2.50 (−3.65, −1.34)	<0.0001	94	−2.88 (−4.18, −1.59)	<0.0001

b: beta, CCS: Complete case scenario.

**Table 8:** Mean changes in weight and BMI post-intervention per groups of the 10 candidate variants in the overall sample.

Variable	Homozygotes for the Non-Effect Allele		Heterozygotes		Homozygotes for the Non-Effect Allele		
	N	Mean (SD)	N	Mean (SD)	N	Mean (SD)	*p* *
Weight Change							
rs6548238_C	2	5.5 (2.12)	10	4.7 (4.64)	71	2.9 (3.35)	0.161
rs1801282_G	2	0.5 (2.12)	4	1 (2.94)	77	2.99 (3.60)	0.310
rs2241766_G	14	1.86 (3.68)	47	2.96 (3.79)	22	3.18 (2.99)	0.575
rs925946_T	66	2.61 (3.73)	14	3.36 (2.79)	3	5.33 (2.08)	0.237
rs1421085_C	10	4.2 (3.82)	21	4.19 (3.89)	52	2.02 (3.17)	0.036
rs1121980_A	10	4.2 (3.82)	23	3.96 (3.80)	50	2.04 (3.23)	0.056
rs17817449_G	10	4.2 (3.82)	23	3.70 (4.16)	50	2.16 (3.09)	0.091
rs3751812_T	8	2.5 (3.30)	23	2.91 (3.30)	52	2.85 (3.76)	0.876
rs9939609_A	10	4.2 (3.82)	21	3.29 (4.39)	52	2.38 (3.09)	0.246
rs17782313_C	2	7 (1.41)	17	4.17 (4.00)	64	2.34 (3.33)	0.043
BMI Change							
rs6548238_C	2	1.50 (0.69)	10	1.66 (1.50)	70	0.86 (1.18)	0.411
rs1801282_G	2	0.27 (0.98)	4	0.26 (1.09)	76	1.04 (1.24)	0.327
rs2241766_G	14	0.70 (1.33)	46	1.03 (1.31)	22	1.07 (1.03)	0.381
rs925946_T	65	0.91 (1.30)	14	1.22 (0.99)	3	1.66 (0.44)	0.238
rs1421085_C	10	1.36 (1.34)	21	1.42 (1.27)	51	0.73 (1.15)	0.119
rs1121980_A	10	1.36 (1.34)	23	1.35 (1.24)	49	0.74 (1.17)	0.125
rs17817449_G	10	1.35 (1.35)	23	1.25 (1.37)	49	0.79 (1.13)	0.163
rs3751812_T	8	0.86 (1.17)	22	1.07 (1.20)	52	0.97 (1.28)	0.744
rs9939609_A	10	1.35 (1.35)	21	1.09 (1.43)	51	0.87 (1.13)	0.212
rs17782313_C	2	2.32 (0.05)	17	1.44 (1.32)	63	0.82 (1.18)	0.063

BMI: Body Mass Index, SD: Standard Deviation. *: *p*-value deriving from the Kruskal-Wallis tests.

## Data Availability

The data presented in this study are available on request from the corresponding author. The data are not publicly available due to participants’ privacy and ethical restrictions.

## Supplementary Materials

The following supporting information can be downloaded at: https://www.mdpi.com/article/10.3390/nu16172842/s1, Figure S1: Convergence checks for MCMC method (Method 2); Table S1: Multivariate linear regressions between the 10 examined SNPs and imputed weight 10 loss post-intervention in the overall sample.
