# Supplementary material for: A Dietary Intervention in Adults with Overweight or Obesity Leads to Weight Loss Irrespective of Macronutrient Composition
Source: Nutrients. 2024 Aug 25;16(17):2842. doi: 10.3390/nu16172842 (PMC11397491; doi:10.3390/nu16172842)
Supplement: Supplementary file 1 [file nutrients-16-02842-s001.zip › nutrients-3161182-supplementary.pdf]

Article

A dietary intervention in adults with overweight and obesity leads to weight loss irrespective of macronutrient composition.

Maria Kafyra [1], Ioanna Panagiota Kalafati [1,2], Garyfallia Stefanou [3], Georgia Kourlaba[4], Panagiotis Moulos[5], Iraklis Varlamis [6], Andriana

Supplemetary Material

Figure S1. Convergence checks for MCMC method (Method 2)

	**Trace plot**	**Autocorrelation plot**
Sc.1	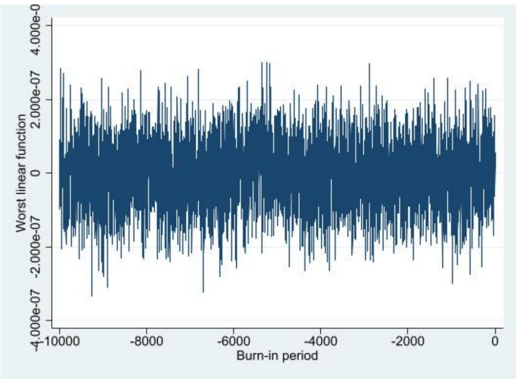	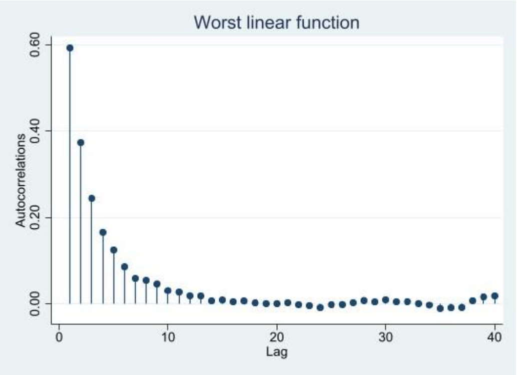
Sc.2	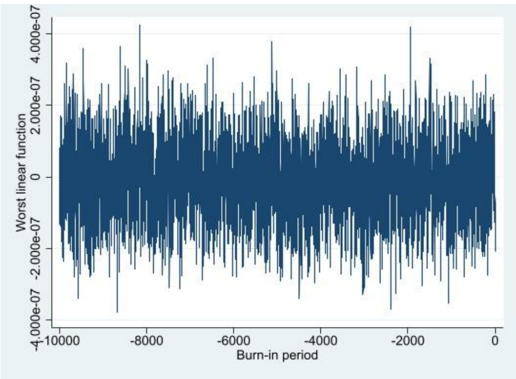	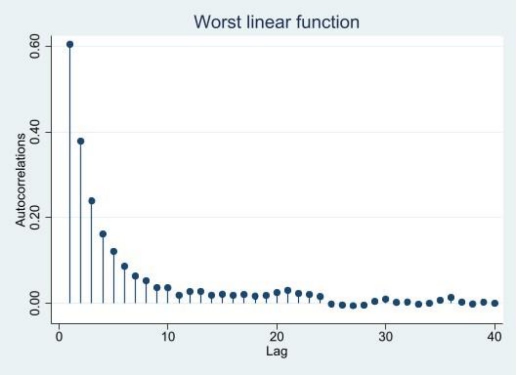
Sc.3	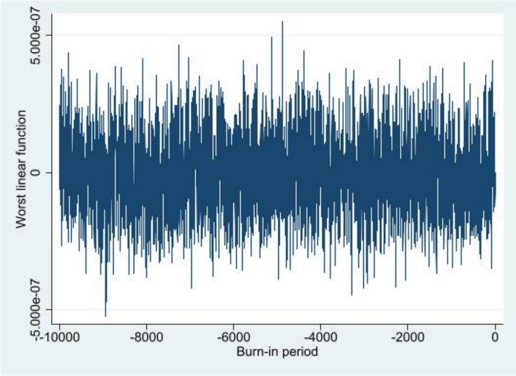	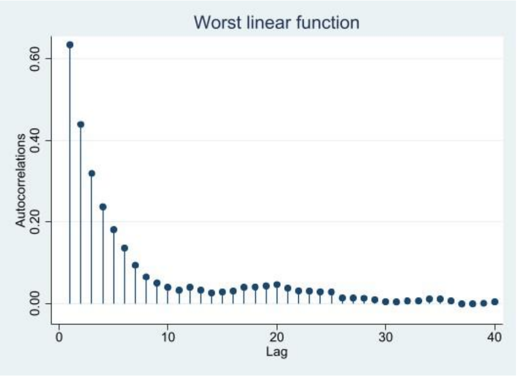

	Trace plot	Autocorrelation plot
Base case	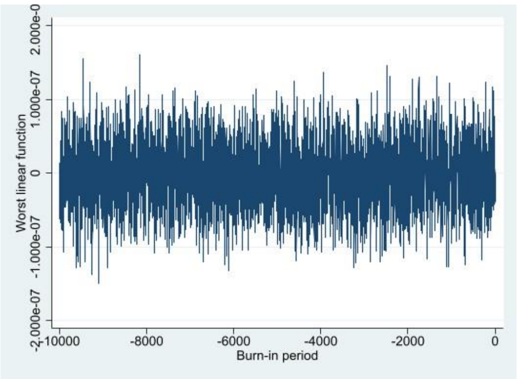	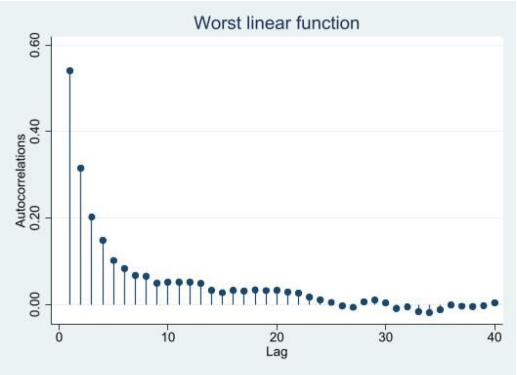

Table S1. Multivariate linear regressions between the 10 examined SNPs and imputed weight loss post-intervention in the overall sample.

	**Model 1**			**Model 2**			**Model 3**		
	Coef*	***SE***	***p***	***Coef****	***SE***	***p***	***Coef****	***SE***	***p***
Weight change									
rs6548238_C									
Heterozygote	0.284	2.901	0.922	1.158	3.001	0.700	1.078	3.005	0.720
Homozygote	1.716	2.758	0.535	1.158	3.001	0.700	2.468	2.886	0.391
rs1801282_G									
Heterozygote	-2.658	4.007	0.509	-2.780	3.996	0.489	-2.727	3.999	0.498
Homozygote	-3.874	3.706	0.299	-4.048	3.709	0.668	-3.962	3.718	0.290
rs2241766_G									
Heterozygote	-0.163	1.122	0.885	-0.168	1.121	0.881	-0.135	1.122	0.904
Homozygote	-0.486	1.275	0.707	-0.431	1.288	0.739	-0.409	1.285	0.751
rs925946_T									
Heterozygote	0.850	1.068	0.427	0.889	1.061	0.404	0.902	1.059	0.396
Homozygote	-0.878	2.193	0.690	-1.129	2.194	0.608	-1.177	2.194	0.593
rs17817449_G									
Heterozygote	-0.077	1.326	0.953	-0.307	1.382	0.824	-0.294	1.381	0.832
Homozygote	0.298	1.262	0.813	0.131	1.312	0.921	1.135	1.311	0.918
rs3751812_T									
Heterozygote	-0.722	1.463	0.623	-0.551	1.441	0.703	-0.388	1.445	0.789
Homozygote	-0.544	1.343	0.686	-0.327	1.341	0.808	-0.145	1.362	0.916
rs17782313_C									
Heterozygote	-1.809	3.404	0.597	-2.353	3.522	0.506	-2.456	3.535	0.490
Homozygote	-0.591	3.325	0.859	-1.157	3.459	0.739	-1.233	3.469	0.723
rs9939609_A									
Heterozygote	0.089	1.394	0.949	-0.105	1.442	0.942	-0.086	1.442	0.952
Homozygote	0.237	1.267	0.852	0.481	1.324	0.971	0.068	1.325	0.959
rs1421085_C									
Heterozygote	-0.309	1.310	0.814	-0.291	1.326	0.827	-0.318	1.325	0.811
Homozygote	0.498	1.147	0.665	0.485	1.174	0.680	0.466	1.173	0.692
rs1121980_A									
Heterozygote	-0.246	1.298	0.850	-0.219	1.311	0.867	-0.238	1.309	0.856
Homozygote	0.502	1.148	0.299	0.479	1.174	0.684	0.458	1.173	0.697

*Coef: β effect size

Model 1: Adjusted for age, sex; Model 2: Adjusting for age, sex, PAL, smoking; Model 3: Adjusting for age, sex, PAL, smoking and diet group.
